# MSC-derived exosomes promote recovery from traumatic brain injury via microglia/macrophages in rat

**DOI:** 10.18632/aging.103692

**Published:** 2020-09-23

**Authors:** Yunfei Chen, Jing Li, Baitao Ma, Na Li, Shihua Wang, Zhao Sun, Chunling Xue, Qin Han, Junji Wei, Robert Chunhua Zhao

**Affiliations:** 1 Center of Excellence in Tissue Engineering, Institute of Basic Medical Sciences and School of Basic Medicine, Chinese Academy of Medical Sciences and Peking Union Medical College, Beijing Key Laboratory of New Drug Development and Clinical Trial of Stem Cell Therapy, Beijing 100005, China; 2 Department of Vascular Surgery, Peking Union Medical College Hospital, Chinese Academy of Medical Sciences and Peking Union Medical College, Beijing 100005, China; 3 Department of Oncology, Peking Union Medical College Hospital, Chinese Academy of Medical Sciences and Peking Union Medical College, Beijing 100005, China; 4 Department of Neurosurgery, Peking Union Medical College Hospital, Chinese Academy of Medical Sciences and Peking Union Medical College, Beijing 100005, China

**Keywords:** human adipose-derived mesenchymal stem cells, microglia, exosomes, traumatic brain injury, neurogenesis

## Abstract

Traumatic brain injury (TBI) is a leading cause of morbidity and mortality in young individuals worldwide. There is currently no effective clinical treatment for TBI, but mesenchymal stem cell-derived exosomes have exhibited promising therapeutic effects. In this study, we performed intracerebroventricular microinjection of human adipose mesenchymal stem cell (hADSC)-derived exosomes (hADSC-ex) in a weight-drop-induced TBI rat model. We found that hADSC-ex promoted functional recovery, suppressed neuroinflammation, reduced neuronal apoptosis, and increased neurogenesis in TBI rats. The therapeutic effects of hADSC-ex were comparable to those of hADSC. Sequential *in vivo* imaging revealed increasing aggregation of DiR-labeled hADSC-ex in the lesion area. Immunofluorescent staining of coronal brain sections and primary mixed neural cell cultures revealed distinct overlap between CM-DiI-labeled hADSC-ex and microglia/macrophages, indicating that hADSC-ex were mainly taken up by microglia/macrophages. In a lipopolysaccharide-induced inflammatory model, hADSC-ex suppressed microglia/macrophage activation by inhibiting nuclear factor κB and P38 mitogen-activated protein kinase signaling. These data suggest that hADSC-ex specifically enter microglia/macrophages and suppress their activation during brain injury, thereby inhibiting inflammation and facilitating functional recovery. They also offer new insight into the cellular targeting, uptake and migration of hADSC-ex, and provide a theoretical basis for new therapeutic strategies for central nervous system diseases.

## INTRODUCTION

Traumatic brain injury (TBI) is a leading cause of death and disability among individuals under 45 years of age [1], and is a known risk factor for chronic neurodegenerative diseases such as Alzheimer's disease and Parkinson’'s disease [2–4]. Patients who survive TBI experience severe neurological and behavioral deficits, along with substantial economic and mental burdens [5–7]. Despite recent progress in TBI research, an effective clinical treatment strategy has not been identified.

Stem cell-based therapies have exhibited great potential in the treatment of TBI. Although stem cell transplantation has significantly ameliorated TBI in animal models and attracted considerable research interest, most patients in clinical practice have not received stem cell therapy [8]. This could be due to poor understanding of the migration, implantation and subsequent integration of transplanted human stem cells into the target brain circuit, as well as concerns about the safety of stem cell treatment [9, 10]. Recent research has indicated that only a small proportion of implanted stem cells differentiate into neural cells after their transplantation into the nervous system, so it is likely that their therapeutic effects are mainly induced by soluble paracrine factors. Therefore, exosomes released by stem cells could be the primary determinants of the beneficial effects of stem cell therapy [10].

Exosomes are 30 to 120 nm extracellular vesicles containing proteins, lipids and nucleic acids, which are secreted by multivesicular endosomes or multivesicular bodies. Exosomes are typically internalized through phagocytosis or endocytosis, but can also fuse with target cell membranes to deliver their contents into the cytosol, where they alter the physiological state of the recipient cell [11]. Thus, exosomes derived from stem cells could be a promising alternative to cell-based therapies. Exosomes are also a safer choice than stem cells because they have lower immunogenicity and no associated ethical issues.

Previous studies have verified the efficacy of mesenchymal stem cell (MSC)-derived exosomes in TBI animal models [12, 13]. Their mechanism of action remains unclear, but could be due to their suppression of inflammatory responses after injury. Microglia are crucial for the activation and regulation of neuroinflammation, and either promote tissue repair or increase tissue damage, depending on their phenotypic polarization (classic [M1, proinflammatory] or alternative [M2, anti-inflammatory]) [14]. Thus, exosomes could ameliorate TBI by altering microglial function *in vivo*, although this has not yet been clearly demonstrated. Furthermore, visualization and *in vivo* tracking studies have demonstrated that only a small proportion of exosomes pass through the blood-brain barrier, suggesting that intravenous administration may not be an optimal method of delivering exosomes to the central nervous system (CNS) [15, 16].

In the present study, we intracerebroventricularly injected human adipose mesenchymal stem cell (hADSC)-derived exosomes (hADSC-ex) into a weight-drop-induced TBI rat model, and examined the sensorimotor functional recovery, neuroinflammation, neuronal apoptosis and neurogenesis in these rats. We also dynamically tracked the biodistribution of hADSC-ex in rat brain ventricles and assessed the cellular uptake of hADSC-ex *in vivo* and *in vitro* to determine their therapeutic mechanism.

## RESULTS

### Intracerebroventricular administration of hADSC-ex facilitated sensorimotor functional recovery in TBI rats

We first obtained and characterized hADSC. We found that hADSC exhibited a characteristic fibroblast-like morphology (Supplementary Figure 1A) and could differentiate into adipocytes or osteoblasts under specific culture conditions (Supplementary Figure 1B, 1C). The cells expressed high levels of CD29, CD44, CD90, CD105 and human leukocyte antigens A, B and C (HLA-ABC), but were negative for CD31, CD34, CD144 and human leukocyte antigen DR (HLA-DR) (Supplementary Figure 1D), as previously reported [17]. We then extracted hADSC-ex and evaluated them using transmission electron microscopy, nanoparticle tracking analysis and Western blotting. The hADSC-ex had a cup-like morphology with a lipid bilayer membrane structure (Supplementary Figure 1E) and a peak diameter of around 100 nm (Supplementary Figure 1F), and expressed exosomal markers such as heat-shock protein 90 (Hsp90), Hsp70, tumor susceptibility gene 101 (Tsg101) and CD63 (Supplementary Figure 1G).

**Figure 1:**
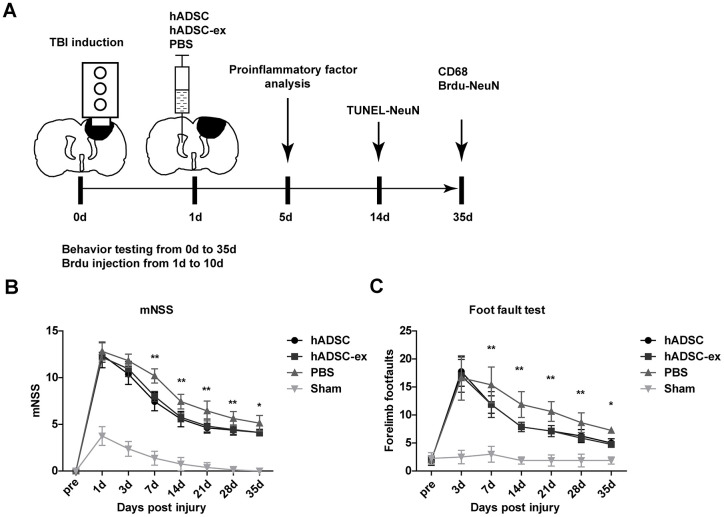
**hADSC-ex facilitated sensorimotor functional recovery in TBI rats.** (**A**) Schematic representation of the experimental procedures. (**B**) Sensorimotor function measured with the mNSS. (**C**) Left forelimb foot-fault test scores. Data represent the mean ± SD, n = 8 rats per group; ns. p > 0.05, * p < 0.05, ** p < 0.01, determined by repeated-measures two-way ANOVA vs. PBS control group.

To study the therapeutic effects of hADSC-ex on TBI, we established a rat model of weight-drop-induced closed head injury, and intracerebroventricularly injected hADSC, hADSC-ex or phosphate-buffered saline (PBS) into the contralateral ventricle 24 h post-injury (Figure 1A). The rats' sensorimotor function was assessed with the modified neurological severity score (mNSS) and a foot-fault test. The mNSS was close to 12 in all rats on day 1 post-TBI, indicating that the neurological deficits in all the TBI rats were comparable before treatment. The mNSS declined gradually in PBS-treated animals between days 3 and 35 post-TBI, indicating that sensorimotor function recovered spontaneously after TBI. However, the functional recovery between days 7 and 35 was significantly faster in the hADSC and hADSC-ex groups than in the PBS-treated control group (Figure 1B). Treatment with hADSC and hADSC-ex also dramatically reduced the frequency of forelimb foot-faults compared with PBS treatment (Figure 1C). Thus, hADSC-ex treatment promoted sensorimotor functional recovery in a TBI rat model, and its therapeutic efficacy was comparable to that of hADSC.

### Intracerebroventricular administration of hADSC-ex inhibited neuroinflammation, reduced neuronal apoptosis and promoted hippocampal neurogenesis

**Figure 2:**
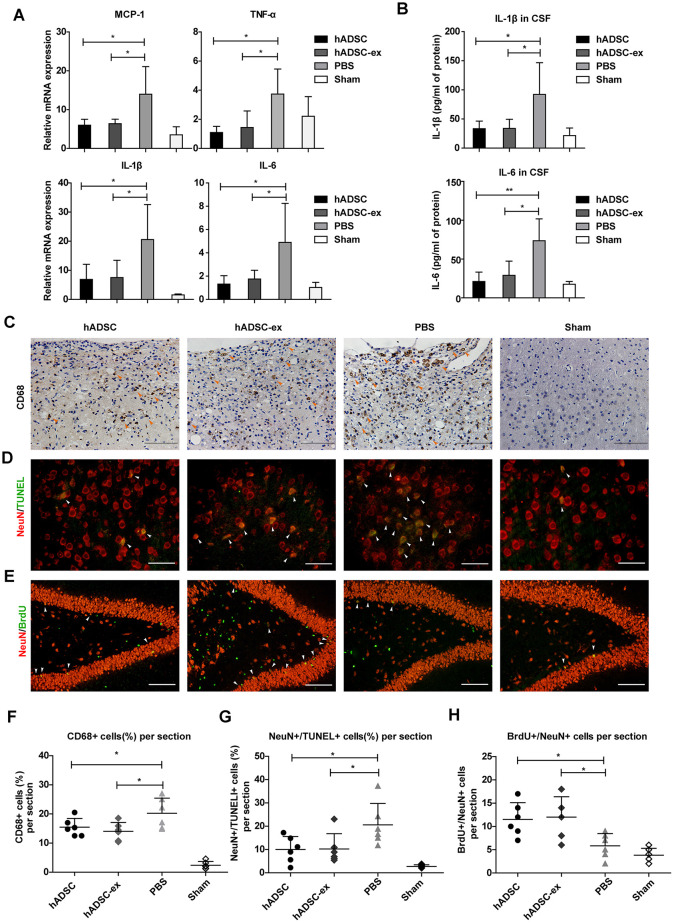
**hADSC-ex suppressed neuroinflammation, reduced neuronal loss in the lesion boundary area and promoted hippocampal neurogenesis.** (**A**) qRT-PCR analysis of proinflammatory factors in damaged brain tissues on day 5. (**B**) ELISA analysis of IL-1β and IL-6 levels in CSF on day 5. (**C**) CD68 immunohistochemical staining for activated microglia/macrophages (indicated by yellow arrows) in the lesion boundary zone on day 35. Scale bar = 100 μm. (**D**) NeuN immunofluorescence staining for mature neurons and TUNEL staining for apoptotic cells in the lesion boundary zone on day 14; double-staining with TUNEL (green)/NeuN (red) for apoptotic neurons is indicated by white arrows. Scale bar = 50 μm. (**E**) NeuN immunofluorescence staining for mature neurons and BrdU staining for cell proliferation in the hippocampal dentate gyrus on day 35; double-staining with BrdU (green)/NeuN (red) for newly generated mature neurons is indicated by white arrows. Scale bar = 100 μm. (**F**– **H**) Scatter plots of data from **C**, **D** and **E**. Data represent the mean ± SD, n = 6 rats per group. ns. p > 0.05, * p < 0.05, determined by one-way ANOVA vs. PBS control group.

Neuroinflammation caused by TBI prolongs secondary brain injury, leading to neuronal cell dysfunction and death [18]. To study the effects of hADSC-ex on neuroinflammation after TBI, we analyzed the expression of proinflammatory factors in injured brain tissues and cerebrospinal fluid (CSF) using quantitative real-time PCR (qRT-PCR) and enzyme-linked immunosorbent assays (ELISAs), respectively, on day 5 after TBI. The mRNA levels of proinflammatory factors (monocyte chemoattractant protein 1 [MCP-1], tumor necrosis factor-alpha [TNF-α], interleukin [IL]-1β and IL-6) in damaged brain tissue (Figure 2A) and the concentrations of IL-1β and IL-6 in CSF (Figure 2B) were significantly lower in the hADSC and hADSC-ex treatment groups than in the PBS control group on day 5 post-TBI.

Long-lasting activation of microglia/macrophages in the chronic phase of TBI impedes neurological functional recovery [19, 20]. To determine the effects of hADSC-ex on sustained microglia/macrophage activation, we performed immunohistochemical staining for the activated microglia/macrophage marker CD68 in paraffin-embedded brain coronal sections on day 35 after TBI. The proportion of CD68+ activated microglia/macrophages in the lesion boundary area was significantly lower in the hADSC and hADSC-ex treatment groups than in the control group (Figure 2C, 2F).

Neuroinflammation exacerbates neuronal loss following TBI. To determine the effects of hADSC-ex on TBI-induced neuronal apoptosis, we performed double-staining with terminal deoxynucleotidyl transferase dUTP nick end labeling (TUNEL, an apoptotic cell marker, green) and NeuN (a mature neuronal marker, red) to identify apoptotic neurons in the lesion boundary area on day 14 after TBI. The proportion of TUNEL/NeuN double-positive cells was lower in the hADSC and hADSC-ex treatment groups than in the PBS control group (Figure 2D, 2G).

Endogenous hippocampal neurogenesis induced by injury [21, 22] is critical for neurological functional recovery following TBI [23, 24]. To elucidate the effects of hADSC-ex on hippocampal neurogenesis, we performed immunofluorescence staining for 5-bromo-2′-deoxyuridine (BrdU, a marker of proliferating cells) and NeuN (a marker of mature neurons) on paraffin-embedded brain coronal sections to identify newly generated neurons on day 35 after TBI. The number of newly generated neurons in the hippocampal dentate gyrus was significantly greater in the hADSC and hADSC-ex treatment groups than in the control group (Figure 2E, 2H). These results indicated that hADSC-ex suppressed acute proinflammatory cytokine production, inhibited chronic microglia/macrophage activation, reduced neuronal apoptosis and promoted hippocampal neurogenesis after TBI. The effects of hADSC-ex were comparable to those of hADSC.

### Visualization and *in vivo* tracking of hADSC-ex after intracerebroventricular administration

**Figure 3:**
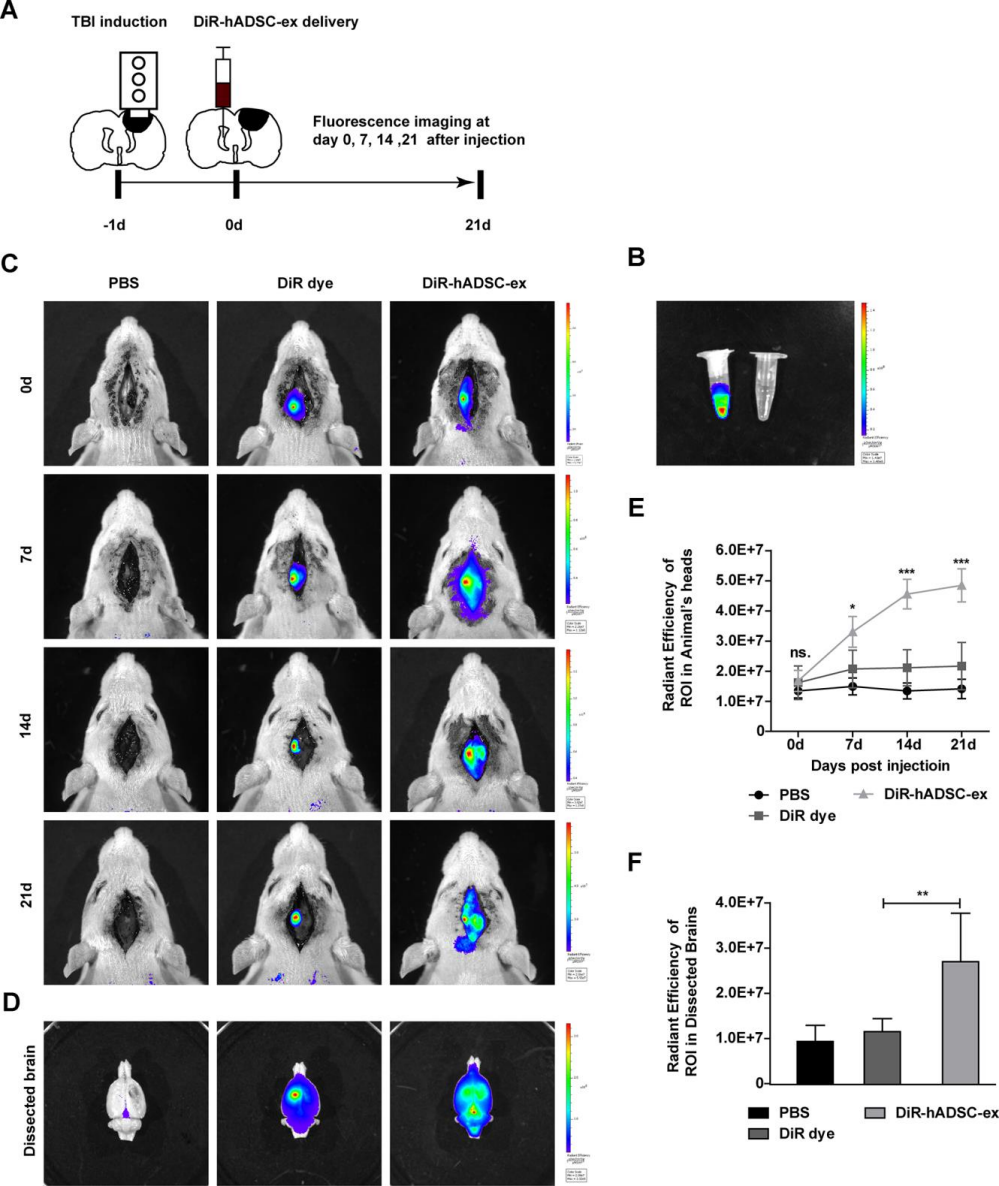
**Visualization and *in vivo* tracking of hADSC-ex after intracerebroventricular administration.** (**A**) Schematic representation of the experimental procedures. (**B**) Representative fluorescence images of DiR-labeled hADSC-ex and PBS. (**C**) Representative fluorescence images of rat heads on days 0, 7, 14 and 21 after administration of PBS, DiR dye or DiR-hADSC-ex. (**D**) Representative fluorescence images of dissected brains on day 21. (**E**) Fluorescence intensity quantification of regions of interest in the lesion sites of rat heads, expressed as the average radiance ± SD, n = 5 rats per group. ns. p > 0.05, * p < 0.05, *** p < 0.001, determined by repeat-measures two-way ANOVA vs. DiR dye control group. (**F**) Fluorescence intensity quantification of regions of interest in the lesion sites of dissected rat brains, expressed as the average radiance ± SD, n = 5 rats per group. ns. p > 0.05, ** p < 0.01, determined by one-way ANOVA vs. DiR dye control group.

To better understand the therapeutic effects of hADSC-ex in TBI rats, we monitored the biodistribution and migration of DiR-labeled hADSC-ex (DiR-hADSC-ex) after their intracerebroventricular administration. The control group was injected with PBS and DiR dye without exosomes (Figure 3A, 3B). Biofluorescence imaging revealed that DiR-hADSC-ex fluorescence in the lesion site accumulated incrementally from day 7 onwards, and was significantly greater than the DiR fluorescence in the control group by day 1 (Figure 3C, 3E). Fluorescence imaging and fluorescence intensity analyses of dissected brain tissues on day 21 confirmed these findings (Figure 3D, 3F). Thus, intracerebroventricularly administered hADSC-ex accumulated in the lesion area in TBI rats. We hypothesized that this phenomenon was due to the uptake of hADSC-ex by certain neural cells, and their subsequent transport to the injury site.

### hADSC-ex were mainly taken up by microglia/macrophages *in vitro* and *in vivo*

**Figure 4:**
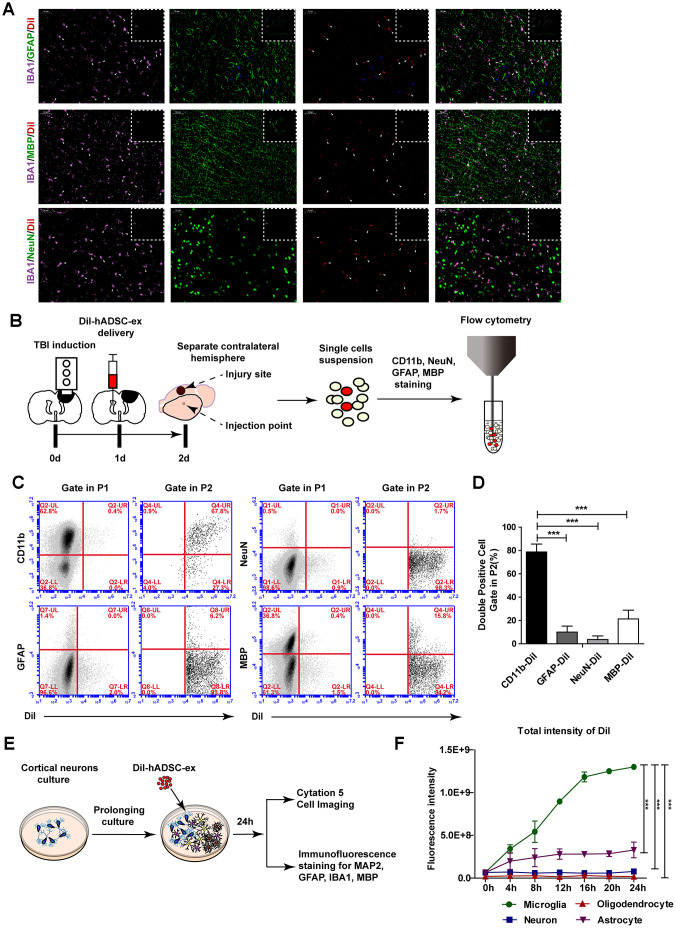
**hADSC-ex were mainly taken up by microglia/macrophages *in vitro* and *in vivo*.** (**A**) Representative images of IBA1/GFAP/DiI, IBA1/MBP/DiI and IBA1/NeuN/DiI immunostaining in the lesion boundary zone in rat brain coronal sections (bregma, −1.5 mm); n = 3, scale bar = 50 μm. The overlapping signals are marked with blue arrows (GFAP/DiI) and white arrows (IBA1/DiI). The white dotted boxes denoted the slices overview and the solid line rectangles indicated the snapshot location. (**B**) Schematic representation of the experimental procedures to detect hADSC-ex cellular uptake by dissociated primary neural cells using FACS. (**C**) Representative dot plots from FACS showing double-positive cell (CD11b/DiI, GFAP/DiI, NeuN/DiI, MBP/DiI) gating in P1 and P2. The gating strategy is shown in Supplementary Figure 3A. (**D**) Bar graphs quantifying the data from (**C**). Data are presented as the mean ± SD, n = 3 independent experiments, *** p < 0.001, determined by one-way ANOVA vs. CD11b/DiI. (**E**) Schematic representation of the use of mixed neural cell cultures to identify the cellular uptake of hADSC-ex *in vitro*. (**F**) Line graph showing the change in the total fluorescence intensity of DiI over time in every neural cell type in the mixed neural cell culture. Data represent the mean ± SD, n = 3 independent experiments, *** p < 0.001, determined by repeated-measures two-way ANOVA vs. microglia.

To determine the mechanism of exosome migration to the lesion area, we labeled hADSC-ex with CM-DiI (DiI-hADSC-ex) and intracerebroventricularly administered them to rats 24 h post-injury. Rat brains were collected on day 21 after the intervention because distinct DiR fluorescence enrichment was observed in the lesion area after 21 days. To determine the cellular location of DiI fluorescence around the lesion boundary zone, we performed immunofluorescence staining for the following markers on frozen brain coronal sections: ionized calcium binding adaptor molecule 1 (IBA1) for microglia/macrophages, glial fibrillary acidic protein (GFAP) for astrocytes, NeuN for mature neurons, and myelin basic protein (MBP) for mature oligodendrocytes. The DiI signals mainly overlapped with those of IBA1, as IBA1/DiI double-positive cells accounted for 88.06% of IBA1+ cells. Weak DiI signals were also observed in some GFAP+ cells, GFAP/DiI double-positive cells accounted for 28.9% of GFAP+ cells. However, the DiI signals scarcely overlapped with those of MBP or NeuN (Figure 4A and Supplementary Figure 2C). Furthermore, there was a higher proportion of microglia/macrophages (IBA1+) with DiI fluorescence signals around the lesion boundary zone than in the corresponding contralateral area (Supplementary Figure 2A, 2B). Thus, hADSC-ex were likely carried to the injury site by microglia/macrophages.

To determine whether microglia/macrophages were the primary neural cells that took up hADSC-ex *in vivo*, we isolated the contralateral hemispheres 24 h after DiI-hADSC-ex administration and dispersed them into single-cell suspensions. The cells were stained with CD11b (a microglia/macrophage marker), GFAP, NeuN or MBP, and the proportion of double-positive cells (CD11b/DiI, GFAP/DiI, NeuN/DiI and MBP/DiI) among DiI+ cells was determined using fluorescence-activated cell sorting (FACS) (Figure 4B). DiI+ cells accounted for 1.1% of all cells (Supplemenatay Figure 3A). CD11b/DiI double-positive cells accounted for 77.63% of DiI+ cells, while GFAP/DiI double-positive cells accounted for 9.1%, NeuN/DiI double-positive cells accounted for 3.6% and MBP/DiI double-positive cells accounted for 22.8% (Figure 4C, 4D). Further, the DiI fluorescence signals in these cells mainly overlapped with those of CD11b, rather than GFAP, NeuN and MBP (Supplementary Figure 3B). Thus, hADSC-ex were mainly taken up by microglia/macrophages *in vivo*.

To confirm that microglia/macrophages were the main neural cells that took up hADSC-ex *in vitro*, we produced mixed neural cell cultures (containing neurons, astrocytes, microglia/macrophages and oligodendrocytes) through the prolonged culture of primary cortical neurons *in vitro*. The mixed neural cells were incubated with DiI-hADSC-ex for 24 h, and the fluorescence intensity of DiI was detected in every type of neural cell for 24 h (Figure 4E). After four hours, the total fluorescence intensity of DiI was significantly greater in microglia/macrophages than in other neural cells (Figure 4F, Supplementary Videos 1–5). Immuno-fluorescence staining confirmed that DiI distinctly overlapped with IBA1 and only weakly overlapped with GFAP, microtubule-associated protein 2 (MAP2) and MBP (Supplementary Figure 2D). These results suggested that hADSC-ex were mainly taken up by microglia/macrophages *in vitro* and *in vivo*.

### hADSC-ex suppressed M1 microglial polarization and promoted M2 microglial polarization *in vitro*

**Figure 5:**
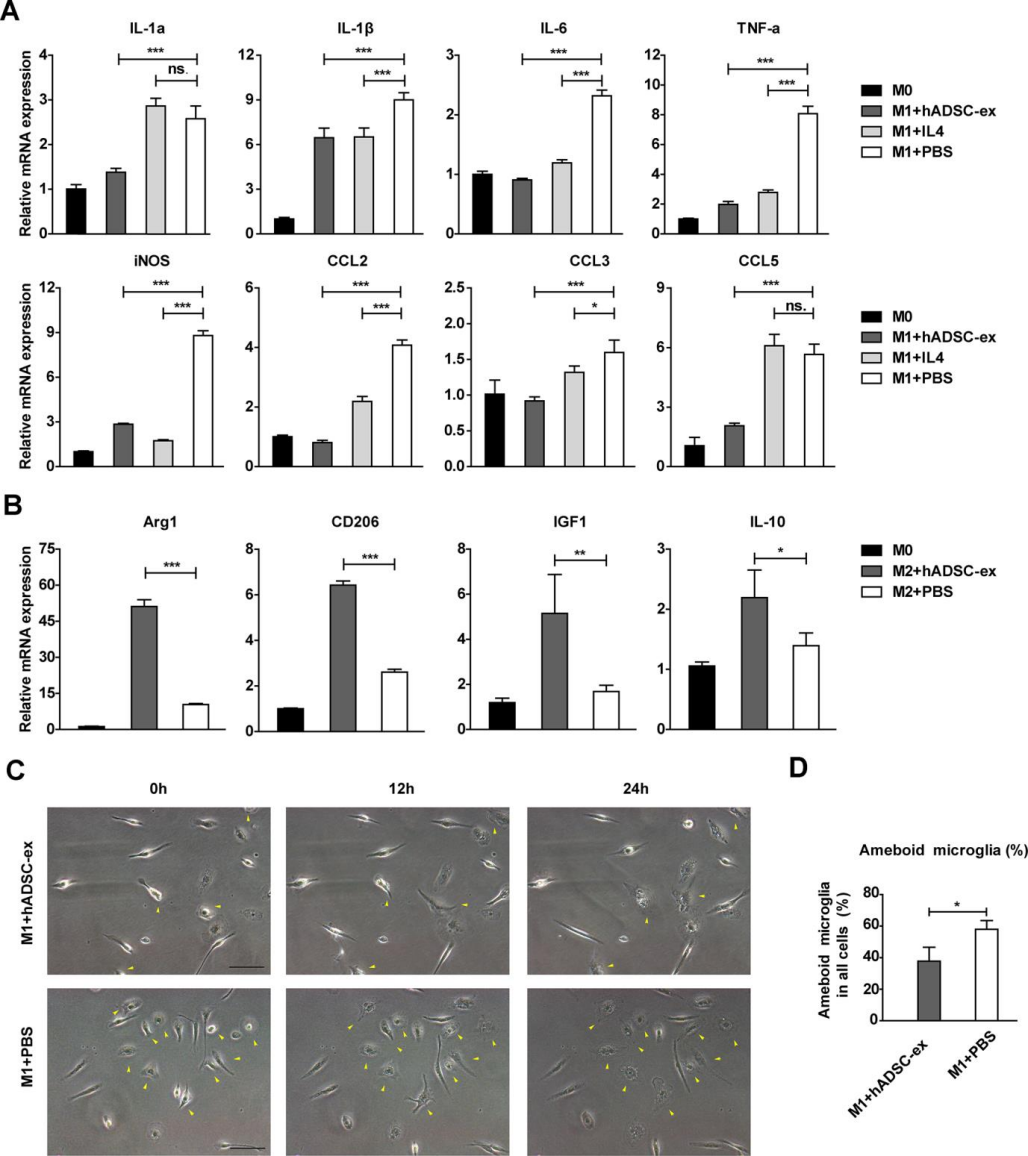
**hADSC-ex alter the functional state of microglia *in vitro*.** (**A**) qRT-PCR analysis of M1-associated factors after culture of M0 microglia in M1 medium (containing 10 ng/mL granulocyte M-CSF, 100 ng/mL lipopolysaccharide and 20 ng/mL interferon-γ) for 12 h with hADSC-ex (200 μg total protein/mL), IL-4 (10 ng/mL) or PBS. Data represent the mean ± SD, n = 3 independent experiments, ns. p > 0.05, * p < 0.05, ** p < 0.01, *** p < 0.001, determined by one-way ANOVA vs. M1+PBS. (**B**) qRT-PCR analysis of M2-associated factors after culture of M0 microglia in M2 culture medium (containing 10 ng/mL M-CSF and 10 ng/mL IL-4) for 12 h with hADSC-ex (200 μg total protein/mL) or PBS. Data represent the mean ± SD, n = 3 independent experiments, ns. p > 0.05, * p < 0.05, ** p < 0.01, *** p < 0.001, determined by one-way ANOVA vs. M2+PBS. (**C**) Representative images of morphological changes in microglia cultured in M1 medium for 24 h; yellow arrows indicate morphological changes. (**D**) The proportion of amoeba-like cells (M1 phenotype) among all cells after culture in M1 medium with hADSC-ex or PBS for 24 h. Data are presented as the mean ± SD, n = 3 independent experiments, * p < 0.05, determined by *t*-test vs. M1+PBS.

To assess the effects of hADSC-ex on the functional status of microglia, we isolated primary microglia from adult rat brains and induced them into different functional states (M0, M1 and M2 phenotypes) as described previously [25, 26]. Microglia were incubated with hADSC-ex during their induction from the M0 to the M1 phenotype. The mRNA levels of M1-associated factors (IL-1α, IL-1β, IL-6, TNF-α, inducible nitric oxide synthase [iNOS], chemokine ligand 2 [CCL2], CCL3 and CCL5) were analyzed using qRT-PCR, and the dynamic morphological changes in microglia were observed using time-lapse live cell imaging, as described previously [27, 28]. The hADSC-ex significantly inhibited the expression of M1-associated proinflammatory cytokines (Figure 5A) and the morphological transformation from the M0 to the M1 state (activated microglia with an amoebic appearance; Figure 5C, 5D). Furthermore, hADSC-ex promoted the mRNA expression of M2-associated cytokines (arginase 1, CD206, insulin-like growth factor 1 and IL-10) in the process of M0 to M2 induction (Figure 5B). Thus, hADSC-ex likely suppressed the microglial functional state change from M0 to M1 and promoted the transition from M0 to M2 *in vitro*.

### hADSC-ex inhibited microglia/macrophage activation by suppressing classical nuclear factor (NF)-kB and mitogen-activated protein kinase (MAPK) signaling

**Figure 6:**
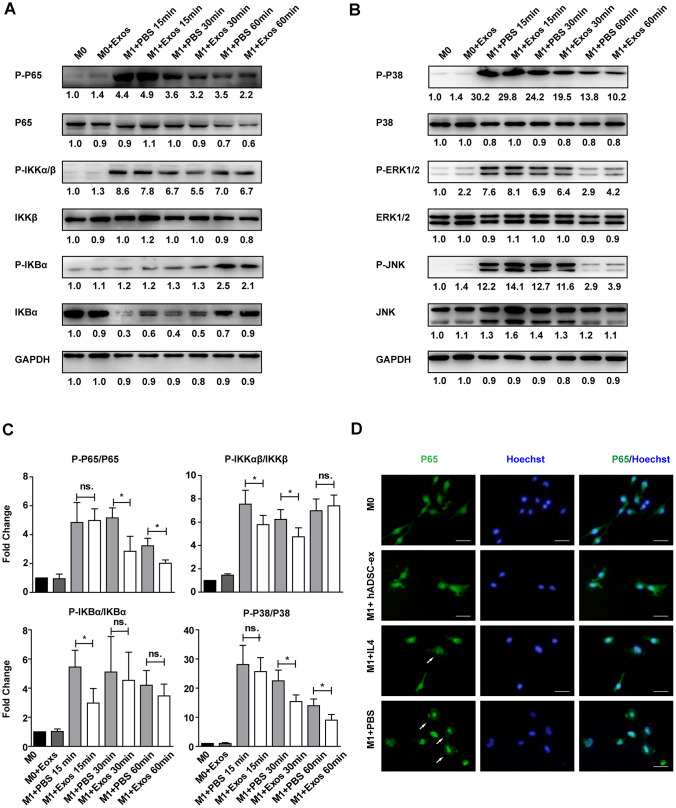
**hADSC-ex suppressed the activation of classical NF-kB and MAPK signaling in primary microglia.** (**A**, **B**) Immunoblots showing P-P65 and P65, P-IKKαβ and IKKβ, P-IKBα and IKBα, P-P38 and P38, P-ERK1/2 and ERK1/2, P-JNK and JNK, and GAPDH in M0 microglia cultured in M0 medium (containing 10 ng/mL M-CSF and 50 ng/mL transforming growth factor β1) or M1 medium, and treated with PBS or hADSC-ex (200 μg/mL) at different time points. (**C**) Fold changes in P-P65 to P65, P-IKKαβ to IKKβ, P-IKBα to IKBα, and P-P38 to P38 levels were each normalized to those of the M0 control group. Data represent the mean ± SD, n = 3 independent experiments, ns. p > 0.05, * p < 0.05, determined by one-way ANOVA vs. M1+PBS. (**D**) M0 microglia were cultured in M1 medium and treated with PBS, IL-4 (10 ng/mL) or hADSC-ex (200 μg/mL) for 24 h. The cells were stained for NF-κB P65 protein (green), and the nuclei were counterstained with Hoechst 33342 (blue). Scale bar = 50 μm. White arrows indicate the enrichment of nuclear NF-κB P65.

The NF-kB and MAPK signaling pathways are important inducers of neuroinflammation and glial cell activation [29–32]. To determine whether hADSC-ex inhibited microglia/macrophage activation by suppressing the NF-κB and MAPK pathways, we assessed the effects of hADSC-ex on the phosphorylation of three NF-kB pathway proteins (P65, inhibitor of NF-kB kinase β [IKKβ] and inhibitor of NF-kB alpha [IKBα]) and three MAPK pathway proteins (P38, extracellular signal-regulated kinase [ERK] ½ and c-Jun N-terminal kinase [JNK]), and on the nuclear translocation of the P65 subunit in primary microglia. The NF-κB and MAPK signaling pathways were markedly activated after M0 microglia were exposed to M1 medium (phosphorylated [P]-P65, P-IKKαβ, P-IKBα, P-P38, P-ERK1/2 and P-JNK levels increased prominently and peaked at different times). However, a significant reduction in P65, IKKαβ, IKBα and P38 phosphorylation was observed in the hADSC-ex treatment group compared with the PBS group (Figure 6A–6C). The phosphorylation of JNK and ERK1/2 did not differ significantly between the hADSC-ex treatment group and the PBS group (data not shown). Immunofluorescence staining revealed signify-cantly greater nuclear localization of NF-κB P65 in the PBS group than in the hADSC-ex treatment group (Figure 6D). These results suggested that hADSC-ex inhibited NF-κB and P38/MAPK activation, thereby downregulating inflammatory molecule expression in microglia.

## DISCUSSION

The poor self-repair capability of the CNS and the complicated pathology of TBI have made it challenging to develop an efficient clinical strategy to prevent and/or treat the secondary injury after TBI. [33]. MSC-derived exosomes have attracted much interest in the treatment of TBI because of their ease of isolation, safety, lack of ethical challenges, and pleiotropic effects. Although the therapeutic effects of MSC-derived exosomes in TBI have already been confirmed [12, 13], the underlying mechanisms have been unclear. In this study, we found that hADSC-ex were mainly taken up by microglia/macrophages and carried to the lesion site after being delivered to the contralateral cerebral ventricle in a rat model of TBI. After their uptake, the hADSC-ex suppressed microglia/macrophage activation, inhibited inflame-matory responses and improved the neural injury microenvironment, thereby ameliorating multiple TBI-induced adverse effects. The beneficial effects of hADSC-ex treatment after TBI included improved sensorimotor functional recovery, reduced neuronal loss and enhanced endogenous hippocampal neurogenesis.

The efficacy of MSC-derived exosomes in the treatment of CNS diseases has been widely reported [34, 35]; however, in most studies, exosomes have been administered intravenously. Although exosomes can pass through the blood-brain barrier [36], *in vivo* biodistribution studies have demonstrated that intravenously administered exosomes mainly accumulate in the liver, spleen, gastrointestinal tract and lungs, while only a small proportion enter the brain, even when modified with rabies virus glycoprotein [15, 16]. Therefore, intravenous administration may not be the optimal exosome delivery method for the treatment of CNS diseases. Patients with moderate to severe TBI usually undergo intraventricular catheter placement for intracranial pressure monitoring because elevated intracranial pressure is associated with poorer outcomes [1]. Exosomes could be administered through intraventricular catheters during intracranial pressure monitoring, thus preventing potential adverse reactions to the systemic distribution of exosomes and improving their therapeutic efficiency. In the present study, we microinjected hADSC-ex into the contralateral lateral ventricle of TBI rats 24 h after injury, thereby reducing the required exosome dosage (20 μg vs. 200 μg required for intravenous administration) [12]. Other routes of exosome administration, such as intrathecal (lumbar) injection or intranasal administration [37], could also be tested. Determining the optimal exosome administration route for the treatment of CNS diseases is an important priority for future research.

The first step of exosome uptake and cargo delivery into acceptor cells is targeting of the acceptor cells. However, it is unclear whether this process is specific or nonspecific and stochastic [38]. We demonstrated that hADSC-ex were mainly taken up by microglia *in vivo* and *in vitro*. Our findings are consistent with those of a recent study indicating that exosomes from oligodendrocytes were internalized preferentially by microglia, rather than by neurons [39]. Thus, MSC-derived exosomes likely specifically enter microglia/macrophages, although we have yet to determine by what pathway they enter.

After their delivery, exosomes have been reported to accumulate at the injury site in a variety of animal disease models [37, 40–44]; however, the mechanisms of exosome migration have been unclear. We also observed exosome accumulation at the injury site. Further, we demonstrated that this phenomenon resulted from the uptake of exosomes by microglia/macrophages and their subsequent transport to the injury site. Microglia are the resident innate immune cells of the CNS, which monitor the microenvironment in the healthy state and respond quickly when brain injury occurs. Activated microglia produce inflammatory molecules such as IL-1β, IL-6, IL-12, TNF-α, metalloproteinases, nitric oxide and reactive oxygen species, which promote inflammatory reactions by increasing the permeability of the blood-brain barrier and facilitating the recruitment of peripheral immune cells [20, 45, 46]. Neuroinflammation initiated by microglial activation is the main cause of secondary injury to the brain, which is the leading cause of aggravated neurological deficits and hospital deaths after TBI [45, 47, 48]. Therefore, normalizing microglial function and inhibiting microglia-induced neuroinflammation could help to prevent or treat neural injury [18]. We found that hADSC-ex suppressed the activation of the NF-kB and MAPK pathways in lipopolysaccharide/interferon-γ-stimulated primary microglial cells (M1 phenotype), prevented the secretion of inflammatory factors and promoted the polarization of microglia to the anti-inflammatory (M2) phenotype. Our results indicated that MSC-derived exosomes mainly entered microglia and exerted anti-inflammatory effects by inhibiting M1 macrophage activation. Although these results may not fully reflect *in vivo* changes in the functional state of microglia, they provide valuable insights into the effects of MSC-derived exosomes on microglial function.

The advantages of exosome-based therapy have been extensively discussed [33, 49]. However, there are a few challenges associated with their routine use in clinical practice: (i) Due to the low yield of exosomes with currently available technologies, large numbers of stem cells are needed to produce therapeutic doses of exosomes. Thus, cell-free exosome treatment could be more costly than stem cell-based therapy. Therefore, it is imperative to improve the yield and purity of exosomes. (ii) Stem cell-derived exosomes are thought to have a lower therapeutic risk than stem cell therapies [50]. However, exosomes have specific biological functions and physicochemical molecular characteristics that could have unforeseen effects [50]. (iii) The composition of exosomal cargo is very complicated, and the various exosomal components could exhibit peculiar effects and interactions. The function of each exosomal component needs to be clarified before exosomes are used in clinical treatment. Active components could be increased through alterations of the culture conditions [51, 52] or modifications to exosomes via bioengineering [53, 54] to maximize their therapeutic effects.

In conclusion, we have reported the first direct evidence that hADSC-ex mainly enter microglia and prevent their proinflammatory activation, thereby improving the injury microenvironment, alleviating aggravated neural injury and facilitating recovery following TBI. We have also demonstrated that the migration of exosomes may be due to their uptake by microglia/macrophages, which carry them to the lesion site. Our findings have extended the current understanding of the therapeutic action of exosomes in CNS diseases and provided valuable new insights into the immunoregulatory mechanisms of MSCs.

## MATERIALS AND METHODS

This study and all the experimental procedures were approved by the Institutional Animal Care and Use Committee of the Academic Committee of the Chinese Academy of Medical Sciences and Peking Union Medical College Hospital.

### Isolation, culture and identification of hADSC and hADSC-ex

Human adipose tissue was obtained from donors who underwent liposuction surgery. The hADSC were isolated, cultured and identified as described in our previous study [17]. The hADSC-ex were also extracted according to previously described methods [55].

### Animal experiments and behavioral assessment following TBI

Adult male Sprague-Dawley rats aged six to eight weeks and weighing 300 ± 11 g (Charles River, Beijing, China) were housed in the animal facility of Peking Union Medical College Hospital. The rats were kept under temperature- and humidity-controlled specific-pathogen-free conditions on a 12-hour light-dark cycle. TBI was induced by Feeney's weight-drop method [56], with a 25-g weight fall from a height of 20 cm. The animals were randomly divided into four groups of eight rats each, as follows: hADSC, hADSC-ex, PBS and Sham. The rats received a contralateral intracerebroventricular injection of a 20 μL solution containing either hADSC (5.0 x 10^5^ cells per rat, based on previous reports [57–59]), hADSC-ex (20 μg total protein per rat, 2.0 x 10^10^ particles/mL) or PBS, 24 h after injury (Figure 1A). Rats in the Sham group underwent surgery without injury or treatment. For proliferating cell labeling, BrdU (50 mg/kg) was injected intraperitoneally into rats daily for 10 days, beginning one day after TBI [60]. To determine the effects of hADSC-ex on neurologic deficits after TBI, two investigators who were blinded to the treatment status performed behavioral analyses using the mNSS [61] and foot-fault test [62] before the TBI and on days 1, 3, 7, 14, 21, 28 and 35 post-injury.

### Immunohistochemical and immunofluorescence staining

The rats were anesthetized with an intraperitoneally administered overdose of pentobarbital sodium, and were transcardially perfused with 200 mL of 0.01 M PBS, followed by 200 mL of 4% paraformaldehyde in 0.1 M PBS (pH 7.4). Their brains were removed, immersed in 4% paraformaldehyde for two to four days, and cut into 4-mm-thick coronal blocks (total of four blocks per animal) using a rat brain matrix. The tissues were embedded in paraffin, and a series of 6-μm-thick slides were cut. After being deparaffinized and rehydrated, the brain sections were boiled in 10 mM citric acid buffer (pH 6) for 10 minutes for antigen retrieval. After being washed with PBS, the sections were incubated with 0.3% H_2_O_2_ in PBS for 10 minutes and blocked with 5% bovine serum albumin (BSA) containing 0.3% Triton X-100 at room temperature for one hour. The sections were then incubated with primary antibodies at 4°C overnight. For the negative controls, the primary antibodies were excluded. The sections were washed with PBS five times, incubated with secondary antibodies at room temperature for one hour, counterstained with 4′,6-diamidino-2-phenylindole and examined by fluorescence microscopy. For immunohistochemical staining, sections were incubated with primary antibodies at 4°C overnight, then incubated with biotin-conjugated secondary antibodies followed by an avidin-biotin-peroxidase reagent, visualized with Fast DAB Peroxidase Substrate (Sigma-Aldrich) and counterstained with hematoxylin.

For cell immunofluorescence staining, cells were fixed in 4% paraformaldehyde for 15 minutes and blocked with 5% BSA containing 0.3% Triton X-100 for one hour. The cells were then incubated with primary antibodies at 4°C overnight, and then with secondary antibodies for one hour at room temperature. Nuclei were counterstained with Hoechst 33342. The following primary antibodies were used: NeuN (Abcam, ab177487), CD11b (Abcam, ab1211), GFAP (Abcam, ab4674), MBP (Abcam, ab62631), CD68 (Abcam, 125212), IBA1 (Proteintech, 10904-1-AP), BrdU (Proteintech, 241-1-Ig) and MAP2 (Proteintech, 17490-1-AP).

### TUNEL staining for apoptotic neurons

To detect neuronal cell death *in vivo* in TBI rats, we performed TUNEL staining of 10-μm paraffin-embedded brain coronal sections (bregma, −1.5 mm) using an In-Situ Cell Death Detection Kit, POD (Roche, 11684817910) according to the manufacturer's protocol.

### Cell counting and quantitation

Cell counting and quantitation were carried out by an investigator blinded to the experimental groups. For the analysis of activated microglia/macrophages, CD68+ activated microglia/macrophages were counted in the lesion boundary zone in eight fields of view in each section, and the proportion of CD68+ activated microglia/macrophages relative to all cells was calculated. For neuronal apoptosis analysis, TUNEL/NeuN double-positive cells were counted in the lesion boundary zone in eight fields of view in each section, and the proportion of apoptotic neurons relative to the total number of neuronal cells was calculated. For neurogenesis analysis, in each section, all NeuN/BrdU double-positive cells were counted in the hippocampal dentate gyrus and its subregions, including the subgranular zone, granular cell layer and molecular layer. The percentage of overlapping signals between DiI and IBA1/GFAP in IBA1+ or GFAP+ cells was calculated in the lesion boundary zone in eight fields of view in each section. The proportion of IBA1/DiI double-positive cells among all cells was calculated in the lesion boundary zone or contralateral side in eight fields of view in each section.

### *In vivo* tracking of hADSC-ex

For *in vivo* tracking, hADSC-ex were stained with DiR dye (Invitrogen, D12731) according to the manufacturer's protocol. PBS, DiR dye or DiR-hADSC-ex were microinjected into the contralateral lateral ventricle 24 h after injury. The rats were anesthetized for observation under a Perkin Elmer (Caliper) IVIS Spectrum In Vivo System on days 0, 7, 14 and 21 after injection. Fluorescence images of DiR-hADSC-ex migration and biodistribution throughout the brain ventricle were acquired at an excitation wavelength of 740 nm and an emission wavelength of 790 nm, and were analyzed using Living Image® 4.5.5 software (Perkin Elmer).

### Flow cytometry analysis of *in vivo* cellular hADSC-ex uptake

CM-DiI dye (Invitrogen, C7000) was used to stain hADSC-ex (DiI-hADSC-ex) according to the supplier's protocol, and the DiI-hADSC-ex were injected into the contralateral brain ventricle of the rats 24 h post-injury. After 24 h, the rats were anesthetized with an intraperitoneally administered overdose of pentobarbital sodium, and were transcardially perfused with PBS. The contralateral hemispheres were immediately extracted, minced and digested with 20 units/mL papain and 0.005% DNase I in PBS. The cell suspension was centrifuged at 200 x *g* for five minutes. Dissociated neural cells were obtained by centrifugation in 0.9 M sucrose in 0.5× Hank's Balanced Salt Solution for 10 minutes at 750 x *g* [63]. The cells were pre-blocked with 5% donkey serum and stained on ice for 30 minutes with optimal concentrations of the primary antibodies and isotype controls. The cells were then washed three times with 3% BSA/PBS, stained with Alexa Fluor 647-conjugated secondary antibodies (Abcam, ab150075, ab150107) in the dark, washed three times and resuspended in 3% BSA/PBS. Cell sorting was performed on a BD Accuri C6 flow cytometer (BD Biosciences), and data were analyzed with Cflow software (BD Biosciences). The following primary antibodies and isotype controls were used: CD11b (Abcam, ab1211), NeuN (Abcam, ab177487), GFAP (Abcam, ab10062), MBP (Abcam, ab62631), rabbit IgG monoclonal-isotype control (Abcam, ab172730) and mouse IgG2a kappa monoclonal antibody-isotype control (Abcam, ab170191).

### Culture of primary adult rat microglia and generation of M0, M1 and M2 phenotype microglia

Primary microglia were isolated from adult male Sprague-Dawley rat brains, as described previously [25]. The cells were cultured (1.2 x 10^5^ cells per well in 2 mL of medium) in six-well poly-d-lysine-coated plates (Sigma-Aldrich, P0296) and grown in microglia culture medium (Dulbecco's modified Eagle's medium/F-12 Glutamax; Gibco, 10565042) supplemented with 10% fetal bovine serum, 100 U/mL penicillin, 100 mg/mL streptomycin and 10 ng/mL rat recombinant carrier-free macrophage colony stimulating factor (M-CSF; Peprotech, 400-28) at 37°C with 5% CO_2_. Half of the medium was changed every three days. M0, M1 and M2 microglia were polarized as described previously [26].

### Primary adult rat mixed neural cell culture and *in vitro* hADSC-ex uptake

Mixed neural cell cultures were obtained through the prolonged culture of purified neurons obtained using OptiPrep™ density gradient (Sigma-Aldrich, D1556) separation. Primary adult rat cortical neurons were isolated from adult male Sprague-Dawley rats, as described previously [64]. Primary cortical neurons were cultured for 14-20 days (until glial cells appeared) to obtain mixed neural cells. To study the cellular uptake of hADSC-ex, we added DiI-hADSC-ex to the mixed neural cells and monitored the changes in DiI fluorescence intensity in each cell type for 24 h using a Cytation 5 Cell Imaging Multi-Mode Reader. The results were analyzed with Gen5 Data Analysis 3.0 Software.

### ELISA

CSF was collected from the rats by means of a needle puncture through the occipito-atlantal membrane on day 5 after TBI. IL-1β and IL-6 protein levels in the CSF were quantified with ELISA kits (Multi Sciences, 70-EK301BHS-96, 70-EK306HS-96) according to the manufacturer's instructions by an investigator blinded to the experimental groups.

### Western blotting

Cells or hADSC-ex were lysed in radioimmuno-precipitation assay lysis buffer (Beyotime, P0013C) with 1 mM phenylmethylsulfonyl fluoride and a protease inhibitor cocktail on ice for 30 minutes. The lysates were sonicated, and proteins were quantified with a BCA Protein Assay Kit (Beyotime, P0012S). Proteins were electrophoretically separated on 10% sodium dodecyl sulfate polyacrylamide gels and electroblotted onto polyvinylidene difluoride membranes (0.22 μm, Millipore, R9AA3602). The membranes were blocked with 5% BSA, incubated with specific antibodies overnight at 4°C, and then incubated with horseradish peroxidase-conjugated secondary antibodies (Proteintech, SA00001-1, SA00001-2) for one hour at room temperature. Protein levels were detected with an enhanced chemiluminescent reagent (Millipore, 345818). The primary antibodies used were: β-actin (Proteintech, 60008-1-Ig), Hsp70 (Proteintech, 66183-1-Ig), Hsp90 (Proteintech, 60318-1-Ig), Tsg101 (Abcam, ab125011), CD63 (Proteintech, 67605-1-Ig), NF-κB Pathway Sampler Kit (Cell Signaling Technology, 9936T), P38 (Cell Signaling Technology, 8690), Phospho-P38 (Cell Signaling Technology, 4511), ERK1/2 (Cell Signaling Technology, 4695), Phospho-ERK1/2 (Cell Signaling Technology, 4370), JNK (Cell Signaling Technology, 9252), Phospho-JNK (Cell Signaling Technology, 9255) and glyceraldehyde 3-phosphate dehydrogenase (GAPDH; Proteintech, 60004-1-Ig).

### RNA extraction and qRT-PCR

Brain tissues or cells were sonicated and lysed with TRIzol (Invitrogen, 10296010). Total RNA was extracted according to the manufacturer's instructions and treated with DNase I (Sigma-Aldrich, AMPD1). Next, cDNA was synthesized using a high-capacity cDNA reverse transcription kit (Takara, RR037Q). The qRT-PCR analysis was performed on a Step-one System (Bio-Rad) with TB Green Mastermix (Takara, R075A). Relative mRNA expression was determined through the 2^–ΔΔCt^ method and normalized to GAPDH expression. The primer sequences are shown in Supplementary Table 1.

### Statistical analysis

Statistical analyses were performed with GraphPad Prism Software 7.0. All data are presented as the mean ± standard deviation (SD), and were analyzed with Student's *t-*test (two groups) or with one-way analysis of variance (ANOVA) or two-way ANOVA followed by Bonferroni's multiple comparison test (more than two groups). Differences between means were considered statistically significant when p was < 0.05. The animal weights were used for randomization and group allocation. No animals were excluded from the analysis.

## Supplementary Material

Supplementary Figures

Supplementary Table 1

Supplementary Video 1

Supplementary Video 2

Supplementary Video 3

Supplementary Video 4

Supplementary Video 5
