# Supplementary material for: MSC-derived exosomes promote recovery from traumatic brain injury via microglia/macrophages in rat
Source: Aging (Albany NY). 2020 Sep 23;12(18):18274–96. doi: 10.18632/aging.103692 (PMC7585083; doi:10.18632/aging.103692)
Supplement: Supplementary Figures [file aging-12-103692-s002..pdf]

SUPPLEMENTARY FIGURES

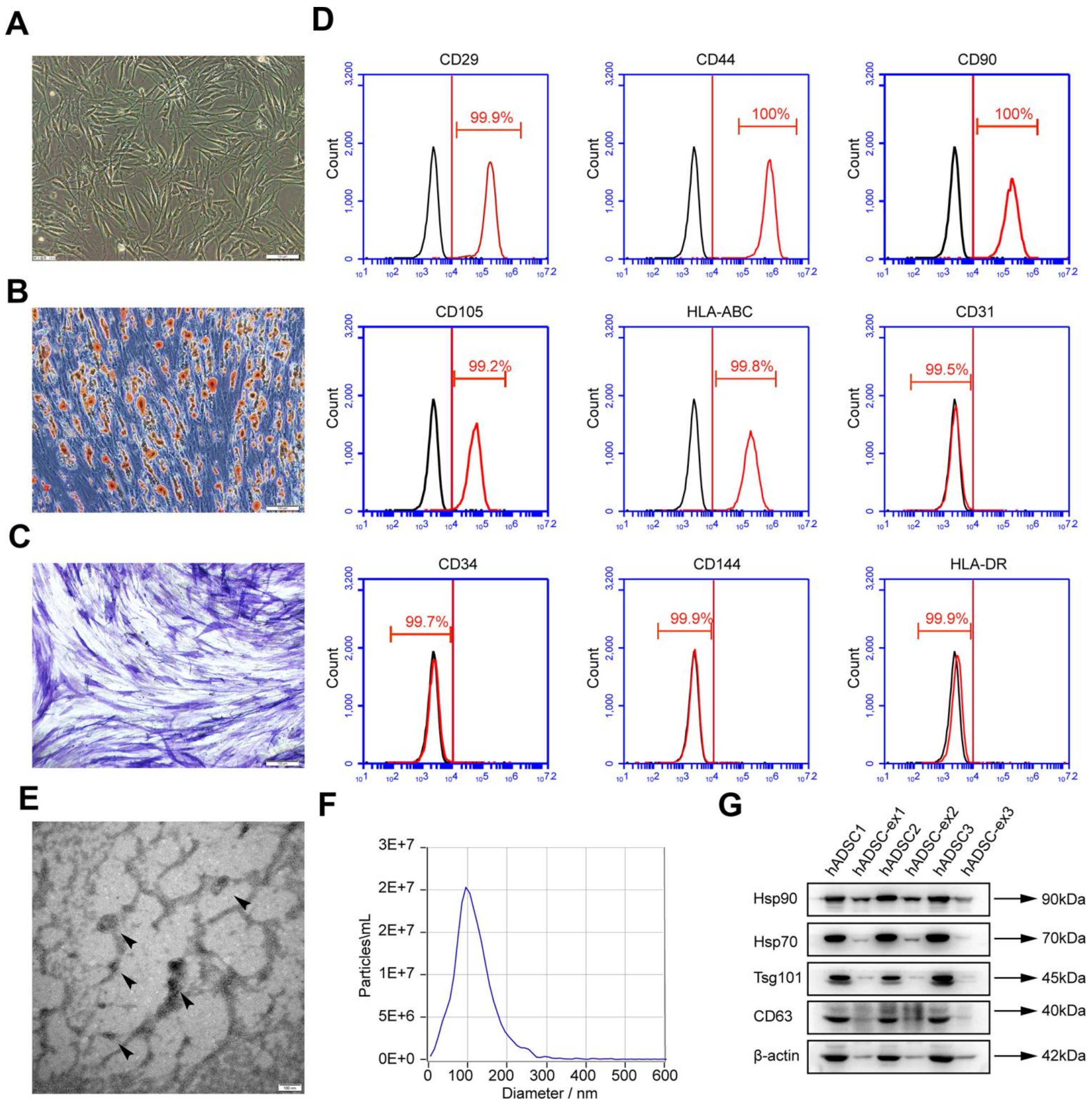

Supplementary Figure 1. Characterization of hADSC and hADSC-ex. (**A**) Cell morphology of hADSC observed under a light microscope. (**B**, **C**) Differentiation capacity of hADSC demonstrated by Oil red O staining for adipocytes and alkaline phosphatase staining for osteoblasts. (**D**) FACS analysis of surface markers on hADSC. (**E**) Representative transmission electron microscopy images of hADSC-ex. Scale bar = 100 nm. (**F**) Size distribution of hADSC-ex, determined with a nanoparticle tracking analyzer. The peak diameter of the particles was 101.4 nm. Concentration = 2.0 x 10^{10} particles/mL. (**G**) Western blot analysis of exosomal markers (Hsp90, Hsp70, Tsg101 and CD63) and β-actin in hADSC and hADSC-ex.

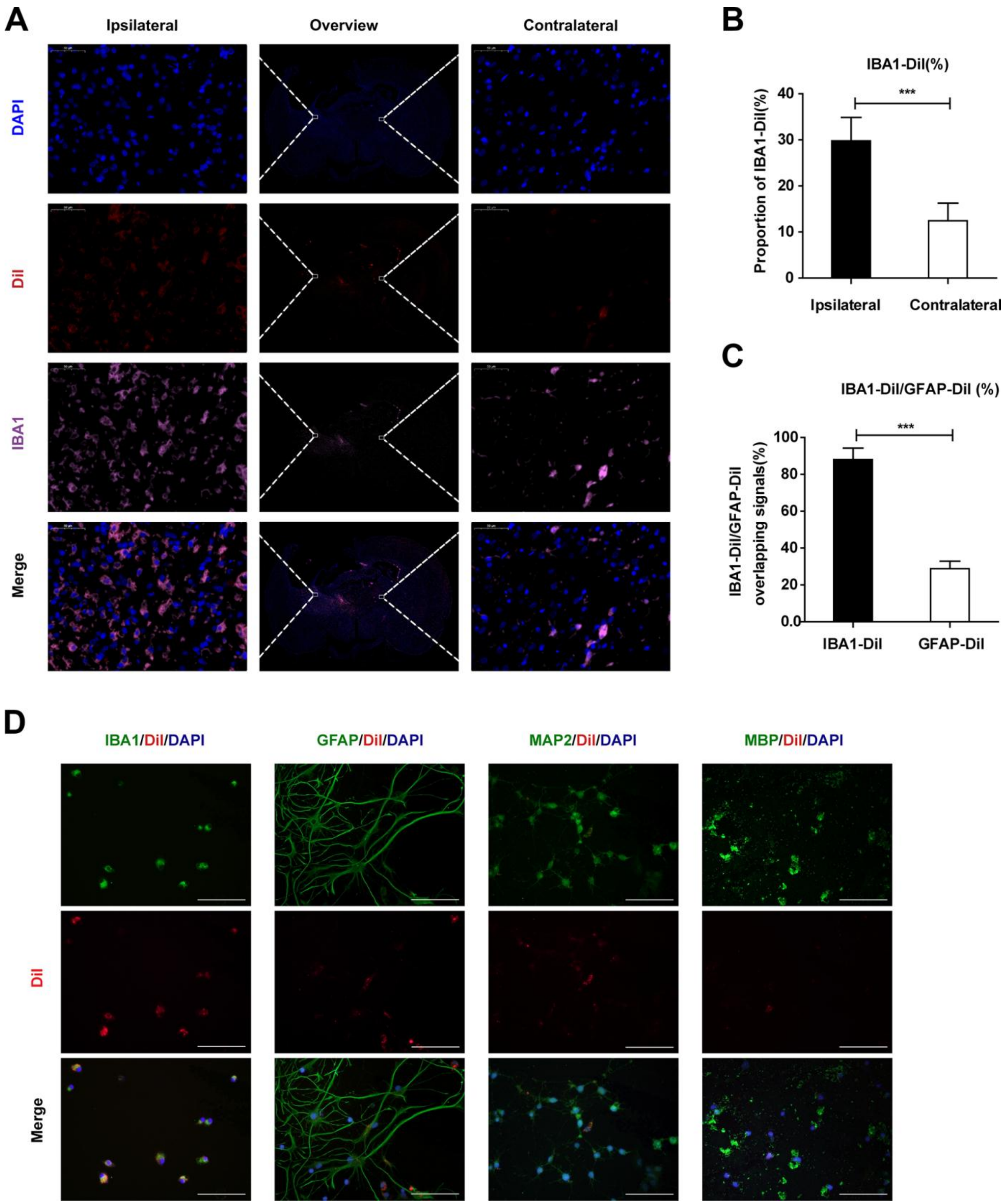

Supplementary Figure 2. hADSC-ex were mainly taken up by microglia/macrophages *in vitro* and *in vivo*. (**A**) Representative images of IBA1/DiI immunostaining in the lesion boundary zone and corresponding contralateral area. (**B**) Quantification of the proportion of IBA1/DiI double-positive cells among all cells in the lesion boundary zone and corresponding contralateral area. Data are expressed as the mean ± SD, n = 3 rats. *** $p < 0.001$, determined by Student's *t*-test. (**C**) Quantification of the percentage of overlapping signals between DiI and IBA1/GFAP in IBA1+ or GFAP+ cells in the lesion boundary zone. Data are expressed as the mean ± SD, n = 3 rats. *** $p < 0.001$, determined by Student's *t*-test. (**D**) Representative images of IBA1, GFAP, MAP2 and MBP immunostaining for microglia/macrophages, astrocytes, neurons and oligodendrocytes, respectively, in the mixed neural cell culture after 24 h DiI-hADSC-ex treatment, to track the cellular uptake of hADSC-ex. Scale bar = 50 μm.

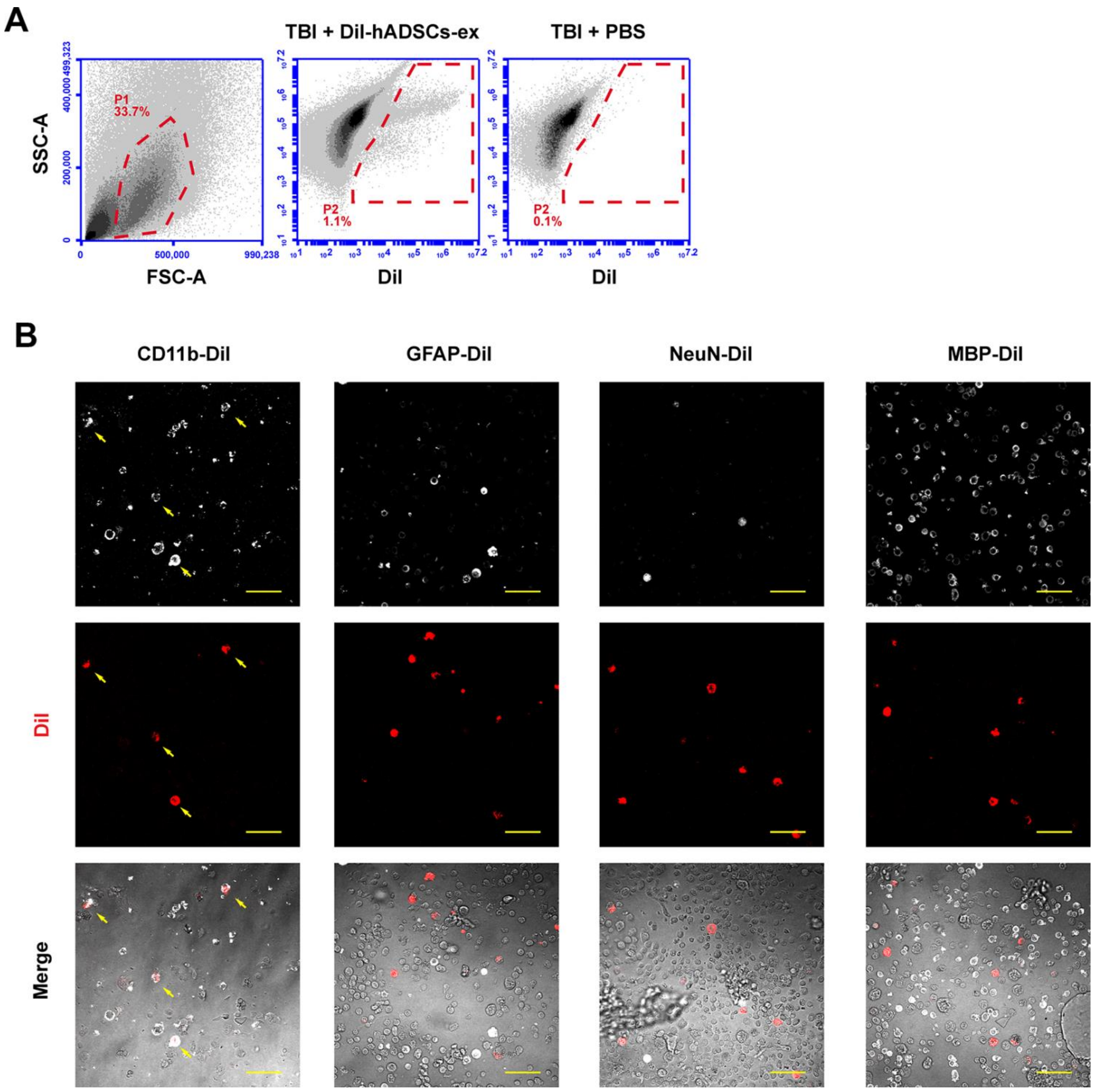

Supplementary Figure 3. hADSC-ex were mainly taken up by microglia/macrophages *in vitro* and *in vivo*. (**A**) Gating strategy for FACS analysis. (**B**) Representative immunostaining images of dissociated primary neural cells under laser scanning confocal microscopy, showing CD11b (white), GFAP (white), NeuN (white), MBP (white) and DiI (red). Scale bar = 50 μm. Double-positive cells are indicated by yellow arrows.
