# Supplementary material for: MSC-derived exosomes promote recovery from traumatic brain injury via microglia/macrophages in rat
Source: Aging (Albany NY). 2020 Sep 23;12(18):18274–96. doi: 10.18632/aging.103692 (PMC7585083; doi:10.18632/aging.103692)
Supplement: Supplementary Table 1 [file aging-12-103692-s001..pdf]

SUPPLEMENTARY TABLE

Supplementary Table 1. Primers used in this study.

Gene	Sense (5′-3′)	Anti-sense (5′-3′)
MCP-1	AACCCTAAGGACTTCAGCACCTT	GCTTGAGGTGGTTGTGGAAAA
TNF-α	TGAACTTCGGGGTGATCGGT	CGCTTGGTGGTTTGCTACGA
IL-1α	GAGGCCATAGCCCATGATTTA	CTCCTGCTTGACGATCCTTATC
IL-1β	AAAAATGCCTCGTGCTGTCT	TCGTTGCTTGTCTCTCCTTG
IL-6	GTTCTCAGGGAGATCTTGGAAATG	GATTGTTTTCTGACAGTGCATCATC
iNOS	AACCCAAGGTCTACGTTCAAG	GCACATCGCCACAAACATAAA
CCL2	GCAGGTCTCTGTCACGCTTC	GGGCATTAACTGCATCTGGCT
CCL3	AACGAAGTCTTCTCAGCGCC	TCTCTTGGTCAGGAAAATGACACC
CCL5	CTCACCGTCATCCTCGTTGC	TTCGAGTGACAAAGACGACTGC
Arg1	GGGTGGAGACCACAGTATGGC	GCATCCACCCAAATGACGCA
CD206	CAACTCTTGGACTCACGGCA	GGGATTCAGCTTCCGGGTTG
IGF1	TACCAAAATGAGCGCACCTCC	GCCTGTGGGCTTGTTGAAGTA
IL-10	AGCTGAAGACCCTCTGGATAC	TGGCCTTGTAGACACCTTTG
GAPDH	AGTGCCAGCCTCGTCTCATA	TGAACTTGCCGTGGGTAGAG
